# Malignant Perivascular Epithelioid Cell Tumor (PEComa) of the Uterus as Part of the Hereditary Cancer Syndrome: A Case Diagnosed with Multiple Malignancies

**DOI:** 10.5146/tjpath.2022.01592

**Published:** 2023-09-15

**Authors:** Sultan Calıskan, Omer Salih Akar, Seda Gun, Mehmet Kefeli

**Affiliations:** Department of Pathology, Ondokuz Mayıs University, Faculty of Medicine, Samsun, Turkey; Department of Genetics, Ondokuz Mayıs University, Faculty of Medicine, Samsun, Turkey

**Keywords:** PEComa, Uterine, Malignant, Breast carcinoma, Colorectal carcinoma

## Abstract

A perivascular epithelioid cell tumor (PEComa) is an uncommon mesenchymal tumor composed of perivascular epithelioid cells. These tumor cells show variable immunoreactivity for both melanocytic and myogenic markers. Occurrence of PEComa has been reported at various anatomical sites, including the gynecological tract, uterus being the most common. Although most patients have sporadic PEComas, a subset may be associated with the inactivation of *TSC1* or *TSC2* genes and the occurrence of *TFE3* gene fusions. However, a relationship between PEComas and other tumors is rare. We report a 41-year-old female patient with malignant PEComa who was admitted to the hospital with a complaint of vaginal bleeding. Because she had previously been diagnosed with colorectal and breast carcinomas at an early age, we performed a comprehensive genetic analysis to identify molecular alterations present in her background that unveiled multiple malignancy predispositions. Next-generation sequencing (NGS) analysis revealed two heterozygous germline pathogenic variants in the *ATM* and *TP53* genes and a heterozygous variant of unknown significance (VUS) in the *BRCA2* gene. The patient was diagnosed with the Li-Fraumeni Syndrome owing to the medical and family history and also the presentation of a pathogenic mutation of the *TP53* gene. There are very few case reports in the literature describing PEComa in the Li-Fraumeni syndrome, and this is the first report of a uterine PEComa in a patient with Li-Fraumeni syndrome.

## INTRODUCTION

Perivascular epithelioid cell tumor (PEComa) is a family of tumors originating from a distinct cell type that is called perivascular epithelioid cells. It has been reported at a wide variety of anatomical locations, including lungs, colon, skin, kidney, bladder, and pancreas, and is also rarely seen in the gynecological tract (1–3). The recent World Health Organization (WHO) tumor classification (2020) defines the perivascular epithelioid cell tumor as “a member of a family of mesenchymal neoplasms composed of perivascular epithelioid cells (PECs) that express melanocytic and smooth muscle markers” (2).

Most of the uterine PEComas show benign/uncertain malignant potential; however aggressive behavior has also been increasingly reported. Some authors have developed classifications based on some features of the tumor to predict outcome in these tumors (2,4–8). In 2005, Folpe et al. analyzed 26 PEComas of the soft tissue and gynecologic tract and suggested some criteria for the classification of these tumors as “benign,” “of uncertain malignant potential,” and “malignant” according to tumor size and histological findings (4). Schoolmeester et al. have analyzed 16 gynecologic PEComas and suggested a modified gynecologic-specific algorithm (5). They proposed making a diagnosis of malignant PEComa based on the presence of at least four of the following features: a size of ≥5 cm, high-grade atypia (excluding degenerative atypia), mitotic rate of > 1/50 HPFs, necrosis, and lymphovascular invasion. Additionally, they suggested reducing the number of categories from three to two categories, namely “benign/uncertain malignant potential” and “malignant.” Bennett et al. analyzed 32 uterine PEComas which constitute the largest series in the literature so far (7). They proposed that a threshold of three (as opposed to four) atypical features more properly classifies a PEComa as malignant. The two proposed algorithms for stratifying the behavior of uterine PEComas were included in the latest version of the WHO tumor classification of female genital tumors (2020) (2).

The tumor group of the PEComa family may be related to genetic alterations of the tuberous sclerosis complex (TSC), an autosomal dominant genetic disease due to loss of function mutations in the *TSC1* (9q34) or *TSC2* (16p13.3) genes, which play a role in the regulation of the Rheb/mTOR/p70S6K pathway (1,6,9). *TFE3* and *RAD51B* gene rearrangements have been also described in a subset of PEComas (2,6,9,10). Because of the sharing of similar genetic features, it is not surprising that tumors associated with TSC can be observed in patients with PEComa as well. However, occurrence of other carcinomas in patients with PEComa is rare. In this report, we describe histopathological and immunohistochemical features of malignant uterine PEComa in a patient who had an early-onset of multiple malignancies, and a family history of recurring cancers. We also performed comprehensive genetic analyses to identify genetic alterations leading to a predisposition to multiple types of cancer.

## CASE REPORT

### Clinical Information

A 41-year-old woman presented with a history of irregular vaginal bleeding for a period of twenty days. She was diagnosed with multiple cancers including metachronous bilateral breast cancer and colorectal cancer, at an early age. At 32 years of age, she was admitted to the hospital when she found a palpable painless mass in her left breast; she was diagnosed with an invasive carcinoma of no special type (ER positive/PR positive/ HER2 negative). Afterwards, she underwent a left segmental mastectomy and was treated with chemotherapy and radiotherapy for 2 years. Three years later, at 35 years of age, she was admitted to the hospital with a complaint of rectal bleeding that had continued for a duration of 4 months. She was diagnosed with colorectal well-differentiated adenocarcinoma and underwent low anterior resection (pT2N0). Furthermore, at 38 years of age, another mass was found on her right breast on the follow-up mammography; she was diagnosed with an invasive carcinoma of no special type (ER negative/PR negative/ HER2 positive). She underwent a right segmental mastectomy and axillary dissection (pT1CN0).

During another follow-up examination, a uterine mass was determined at the corpus posterior and was thought to be leiomyoma on the transabdominal ultrasonography. Magnetic resonance imaging confirmed a 6.3 x 6 cm mass originating from the left side of the uterus and a nearly two-fold increase in the size of the mass compared to ten months prior. Due to the the rapid growth of the mass, she underwent a total abdominal hysterectomy and bilateral salpingo-oophorectomy.

### Histopathologic, Immunophenotypic, and Molecular Findings

**Figure 1:**
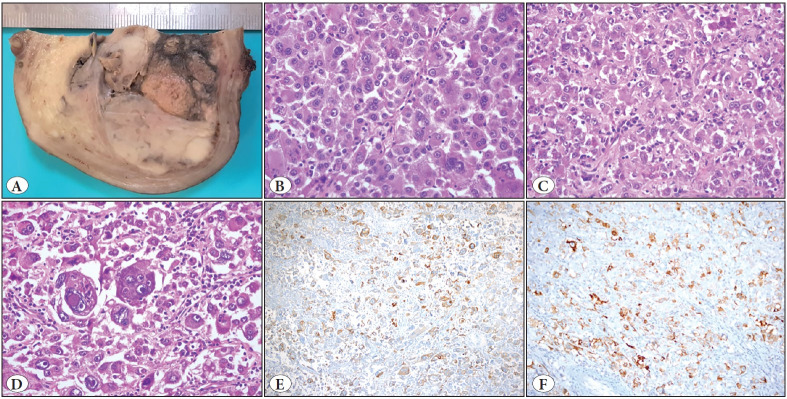
Macroscopic appearance of tumor **(A).** Tumor was composed of discohesive atypical epithelioid cells with inclusion-like eosinophilic nucleoli **(B, C),** binucleated and multinucleated atypical cells **(D)**. Tumor cells expressed HMB-45 **(E)** and Melan A **(F).**

Macroscopically, the uterus was measured as 10 x 7.5 x 7.4 cm and there was a nodular, smooth-bordered, intramural mass arising from the left side of the uterine corpus. The dimensions of the mass were 5 x 4.8 x 4.5 cm. The cut surface of the mass was solid and gray-white in color, and several foci of hemorrhage and necrosis were present (Figure 1). Microscopically, the mass had an expansile border and consisted of single, discohesive atypical epithelioid cells. Cellularity was moderate, and there was a trace amount of intervening and focal hyalinized stroma which was rich in lymphocytes, plasma cells, and thin- and thick-walled blood vessels. Areas of necrosis constituted less than 50% of the tumor. The tumor cells had distinct atypical features characterized by a large size, pleomorphic shape, and an eosinophilic/pale granular cytoplasm with macronuclei. There were many binucleated and multinucleated tumor cells. Most tumor cells had large, inclusion-like eosinophilic nucleoli similar to melanoma cells. Some of the cells had large intranuclear pseudoinclusions (Figure 1). The mitotic count was 15 mitoses/50 HPFs. Lymphovascular invasion was detected. There was a brown-black melanin pigment within the histiocytes.

**Figure 2:**
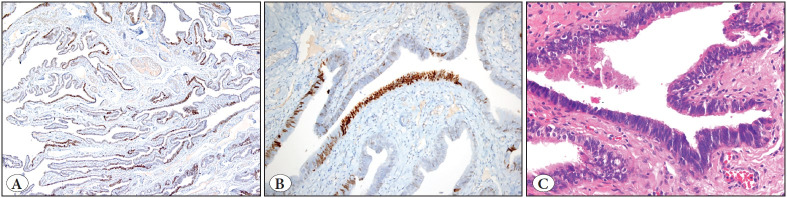
p53 signature of fimbrial epithelium of tuba uterina. Strong nuclear p53 staining in more than 12 cells of fimbrial epithelia at low power **(A),** and high power **(B),** and appearance of the same fimbrial epithelia at hematoxylin-eosin stained section **(C).**

Immunohistochemically, the tumor cells were focally positive for HMB-45 (HMB45, Ventana) (Figure 1), Melan-A (A103, Ventana) (Figure 1), TFE-3 (MRQ-37, Cell Marque), vimentin (V9, Ventana), SMA (1A4, Cell Marque), desmin (DE-R-11, Ventana), caldesmon (E89, Cell Marque), CD-68 (Kp-1, Cell Marque), and progesterone receptor (1E2, Ventana). The tumor cells were negative for cytokeratin (AE1/AE3/PCK26, Ventana), S100 (Ventana), SOX-10 (SP267, Cell Marque), estrogen receptor (Sp1, Ventana), CD10 (EP195, BioSB), GATA-3 (L50-823, Cell Marque), CD61 (2f2, Cell Marque), CD117 (9.7, Ventana), CD45 (RP2/18, Ventana), and cyclin D1 (SP4, Ventana). The Ki67 (GM010, Genemed) index was 30%. The endometrium, cervix, both ovaries and fallopian tubes were tumor-free. However, multiple “p53 (Bp53.11, Ventana) signature” foci were identified in both fimbrial epithelia (Figure 2). The tumor was diagnosed as PEComa based on histopathological and immunohistochemical findings. The tumor was categorized as malignant due to its possessing all the atypical criteria according to WHO classification (2).

**Figure 3:**
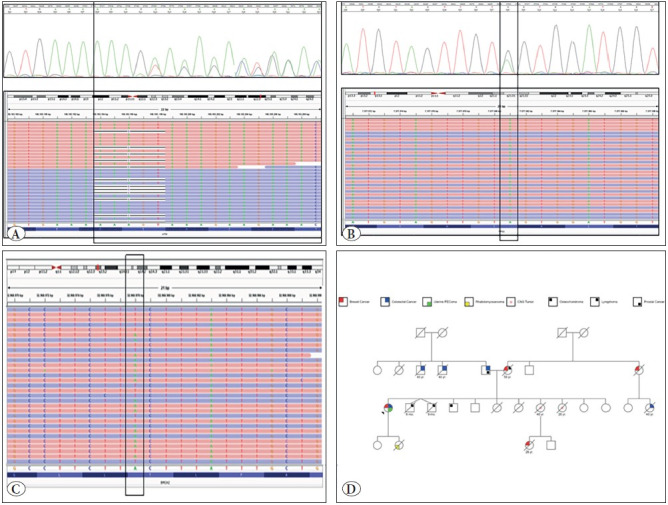
Next-Generation Sequencing analysis revealed a heterozygous 5-bp deletion resulting in a frameshift mutation and a premature stop codon in the ATM gene **(A),** a heterozygous missense mutation in the TP53 gene **(B),** and a missense variant classified as Variant of Unknown Significance (VUS) in the BRCA2 gene **(C).** Sanger sequencing analysis also confirmed both mutations. The family pedigree of the patient **(D).**

Because the patient had a diagnosis of early-onset bilateral breast cancer, colorectal cancer, and the presence of the “p53 signature” in the tubal fimbrial epithelium, genetic testing for germline *BRCA1*-*BRCA2* mutations (BRCA MASTR Plus Dx; Multiplicom, Niel, Belgiumand) with a comprehensive hereditary cancer panel (Qiagen, Hilden, Germany) was performed. Next-Generation Sequencing (NGS) (Illumina, San Diego, CA, USA) analyses with Comprehensive Hereditary Genes Panel (including *ABRAXAS1, AIP, APC, ATM, ATR, AXIN2, BAP1, BARD1, BLM, BMPR1A, BRIP1, BUB1B, CDH1, CDK4, CDKN2A, CHEK2, CTNNA1, EPCAM, FANCC, FLCN, GALNT12, GEN1, GPC3, GREM1, HOXB13, MEN1, MET, MLH1, MRE11, MSH2, MSH6, MUTYH, NBN, NTHL1, PALB2, PALLD, PIK3CA, PMS1, PMS2, POLD1, POLE, PRSS1, PTCH1, PTEN, RAD50, RAD51B, RAD51C, RAD51D, RINT1, SDHB, SDHC, SDHD, SMAD4, SMARCA4, STK11, TP53, VHL, XRCC2, RET, TSC1, TSC2 *genes) and germline Multiplex Ligation Probe Amplification (MLPA) analyses for both *BRCA1* and *BRCA2* genes were performed with patient’s blood. NGS analysis revealed 2 heterozygous germline pathogenic variants in the *ATM *(Figure 3) and *TP53 *(Figure 3) genes, and a heterozygous variant of unknown significance (VUS) in the *BRCA2* gene (Figure 3). The first variant was localized in the *ATM*, namely NM_000051.3(ATM):c.5979_5983delTAAAG (p.Ser1993ArgfsTer23) in exon 40, and the second one was localized in *TP53*, namely NM_001126112.2(TP53):c.700T>C (p.Tyr234His) in exon 7. *BRCA2* gene analysis revealed NM_000059(BRCA2):c.9409A>T (p.Thr3137Ser) in the exon 25. Both ATM:c.5979_5983delTAAAG (p.Ser1993ArgfsTer23) (rs876660134, Clinvar Accession: RCV000219008.5) and NM_001126112.2(TP53):c.700T>C (p.Tyr234His) (rs864622237, RCV000492782.1) are known pathogenic variants for hereditary cancer syndromes (11,12). Germline pathogenic mutations of *TSC1* or *TSC2* genes were not detected.

Because the patient had metachronous early-onset multiple cancers and also had germline pathogenic mutations of *ATM* and *TP53* genes, a detailed family medical history was analyzed by a clinical geneticist. In the family history, her four-year-old daughter had died because of embryonal rhabdomyosarcoma. We learned that some of the other family members of the patient also had cancer diagnoses. The family pedigree of the patient was investigated and shown in Figure 3. The patient was diagnosed with the Li-Fraumeni Syndrome owing to the personal and family history and also the presentation of a pathogenic mutation of the *TP53* gene. She has been followed for two years after the PEComa diagnosis and there was no evidence of recurrence or metastasis.

## DISCUSSION

Perivascular epithelioid cell tumors are rarely seen in gynecological organs, and the uterus is the most common site of involvement (2,6,13). Although most patients had sporadic PEComas, a subset of PEComas (~10%) may be seen within TSC. The most common genetic alterations in sporadic and hereditary PEComas demonstrate inactivation of the *TSC2 (16p13.3)* and the *TSC1 (9q34) *genes. These genes are inactivated, and then subsequently activated by the mammalian target of the rapamycin (mTOR) pathway, providing a potential focus for targetable therapy (3,14). The gene rearrangement of the MIT/TFE family of transcription factors member *TFE3* (chromosome Xp11.23) has been reported in a small minority of PEComas, including *SFPQ/PSF-TFE3* fusion and *DVL2-TFE3* fusion (14,15). *TFE3*-related PEComas share similar histopathological features, such as epithelioid appearances, alveolar or nested growing patterns, and low nuclear atypia (5,6,15,16). *RAD51B* (14q24) translocation, related to *RAD51B-RRAGB* or *RAD51B-OPHN1* gene fusions, was demonstrated in three uterine PEComa patients (10). Zhang et al. found the most frequent somatic mutations in *ATM*, *BRCA2*, and *APC* other than the *TSC* genes in patients with pulmonary lymphangioleiomyomatosis (17). Bing et al. studied p53 expression and gene mutation in pure epithelioid PEComas from the kidneys, heart, liver, and uterus. They found greater p53 expression and mutation in epithelioid angiomyolipomas (18). In a recent study, Akumulla et al. analyzed comprehensive genomic profiling of 31 metastatic PEComas and they detected a total of 100 genomic alterations tumor samples of the series of patients. The genes most commonly altered in that study were *TP53* (45.2%), *TSC2* (32.3%), *RB1* (25.8%), *CDKN2A* (19.3%), *TFE3* (16.1%), *ATRX* (9.6%), *TSC1* (9.6%), and *CD36*, *FLCN*, *NF1* and *SMARCB1* (6.4%, each). *TFE3* rearrangements have also been identified in 16% of the tumors (19).

Our patient had all of the histopathological criteria of the modified gynecologic-specific algorithm and was reported as malignant PEComa. The patient had no *TSC1/TSC2* and *RAD51B* mutations and no clinical TSC findings either. We detected focally TFE3 positivity as per the immunohistochemical protocol (an automatic protocol) that may reveal *TFE3* translocation-associated PEComas. However, we did not confirm this by either FISH or RT-PCR. We found germline pathogenic mutations in *ATM *and *TP53 *genes and a variant of unknown significance in the* BRCA2 *gene, which is clarified by the multiple carcinogenesis background of the patient and indicated in hereditary cancer syndromes, particularly the Li-Fraumeni Syndrome.

The Li-Fraumeni Syndrome (LFS) is a hereditary cancer syndrome associated with germline pathogenic mutations of the *TP53* tumor suppressor gene*. *A wide spectrum of tumors including soft tissue sarcomas, osteosarcomas, early-onset breast cancers, brain tumors, colon cancer, gastric cancer, leukemia, and adrenocortical carcinomas has been associated with this syndrome (20). Classical LFS is diagnosed when a patient has all of the following three criteria; a sarcoma diagnosed before 45 years of age, a first-degree relative with any cancer diagnosed before 45 years of age, a first- or second-degree relative with any cancer diagnosed before 45 years of age or a sarcoma diagnosed at any age (20,21). Tumors seen in the patient’s family members including premenopausal breast cancer, embryonal rhabdomyosarcoma, and tumors of the central nervous system are well-defined cancer types in LFS. Because our patient had all of these criteria in addition to a mutant *TP53 *gene, she was diagnosed with LFS. PEComas located in various sites have also been described in LFS/*TP53* mutation carriers. Neofytou et al. described two synchronous primary PEComa of the liver and the right kidney in a 24-year-old patient with LFS (22). Galera López et al. reported a simultaneous diagnosis of PEComa of the liver in two siblings with an LFS (23). Butz et al. reported a malignant, metastatic PEComa of the thigh muscle harboring a novel *TP53* germline splice mutation in an attenuated LFS patient (24). Our case is the first uterine PEComa with related LFS.

Biallelic germline mutations of the *ATM* gene are associated with the autosomal recessive ataxia-telangiectasia syndrome, characterized by cerebellar degeneration, telangiectasia, immunodeficiency, cancer susceptibility, and radiation sensitivity (25). Carriers of monoallelic pathogenic germline mutations of the *ATM* gene have also been related with varied tumor predisposition, particularly lymphomas and leukemia (26,27). Some tumors that are seen in the patient’s family may be associated with carrying an additional mutant *ATM* gene over the mutant *TP53* gene. However, the patient does not have any neurological symptoms such as ataxia, dysarthria, and postural instability; or other symptoms of the ataxia-telangiectasia syndrome.

## CONCLUSION

Uterine PEComas are very rare tumors and can be associated with syndromes, particularly TSC. Rarely, PEComas may be related to hereditary cancer syndromes other than TSC, and these patients may have other malignancies that have specific gene mutations such as *TP53*. It is important to make a true diagnosis of PEComa for the patient and to screen the family for possible cancers. With respect to family history, genetic analyses and counseling can be helpful for the family’s future assessments.

## Conflict of Interest

The authors declare no conflict of interest.
